# Promising Antidiabetic and Antimicrobial Agents Based on Fused Pyrimidine Derivatives: Molecular Modeling and Biological Evaluation with Histopathological Effect

**DOI:** 10.3390/molecules26082370

**Published:** 2021-04-19

**Authors:** Fatma Bassyouni, Mohammad Tarek, Abeer Salama, Bassant Ibrahim, Sawsan Salah El Dine, Nemat Yassin, Amina Hassanein, Maysa Moharam, Mohamed Abdel-Rehim

**Affiliations:** 1 Chemistry of Natural and Microbial Products Department, Pharmaceutical Industry Research Division, National Research Centre, Cairo 12622, Egypt; fatma.nrc.eg@gmail.com; 2 Bioinformatics Department, Armed Forces College of Medicine (AFCM), Cairo 12622, Egypt; mohammadtareq459@gmail.com; 3 Pharmacology Department, Medical Division, National Research Centre, Cairo 12622, Egypt; berrotec@yahoo.com (A.S.); bmmih1974@gmail.com (B.I.); sawsannrc@yahoo.com (S.S.E.D.); nematyassin@yahoo.com (N.Y.); 4 Pathology Department, Medical Division, National Research Centre, Cairo 12622, Egypt; hassaneinamina@yahoo.com; 5 Chemistry Department of Microbial Products, Biotechnology Research Division, National Research Centre, Cairo 12622, Egypt; maysa_nrc@live.com; 6 Department of Materials and Nanophysics, KTH Royal Institute of Technology, SE11419 Stockholm, Sweden

**Keywords:** molecular modeling, pyrimidine derivatives, antidiabetic activity, antimicrobial activity, histopathological study

## Abstract

Diabetes is the most common metabolic disorder in both developing and non-developing countries, and a well-recognized global health problem. The WHO anticipates an increase in cases from 171 million in 2000 to 366 million by 2030. In the present study, we focus on the preparation of pyrimidine derivatives as potential antidiabetic and antimicrobial agents. Thein vivoeffect on total serum glucose concentration, cholesterol and antioxidant activity was assessed in adult male albino Wister rats and compared to the reference drug glimperide. Promising results were observed for compound **5**. The histopathological study confirms that compound **5** results in significant activity with liver maintenance. The antimicrobial activities were evaluated against several bacterial strains such as *Salmonella typhimurium* ATCC 25566*, Bacillus cereus, Escherichia coli* NRRN 3008*, Pseudomonas aeruginosa* ATCC 10145*, Staphylococcus aureus* ATCC 6538and fungi such as *Rhizopus oligosporus, Mucor miehei* and *Asperillus niger.* Compounds **4** and **5** showed a good inhibition of the bacterial zone compared to the reference drug cephradine. Finally, we suggest protein targets for these drugs based on computational analysis, and infer their activities from their predicted modes of binding using molecular modeling. The molecular modeling for compounds **4** and **5** resulted in improved docking scores and hydrogen bonding. The docking studies are in good agreement with the in vitro and in vivo studies.

## 1. Introduction

Diabetes mellitus has been recognized as one of the most serious public health problems worldwide. About 65–75% of people with diabetes die from the disease. Diabetes mellitus is characterized by hyperglycemia caused by insulin resistance and leads to chronic cardiovascular diseases and kidney diseases known as diabetic nephropathy. Diabetes is a chronic disease resulting from the inability of the pancreas to produce enough insulin. Diabetes increases the risk of heart disease by up to four times [1,2,3].

Pyrimidine derivatives have properties that are potentially useful in combating diabetes. Pyrimidine derivative-containing heterocyclic compounds in combination with other moieties play several roles in biological processes and have significant chemical and pharmacological importance in the industry. In addition, pyrimidines in conjugation with a thiazole nucleus have gained prominence in medical and clinical applications [4]. Thiazolopyrimidines have been gaining popularity in recent years for their outstanding use as components of pharmacological compounds such as bioavailable CXCR2 antagonistand anti-diabetic agents [5], antimicrobial agents [6], antioxidants **[7]**, anti-inflammatory agents [8], antimalarials [9], and antitubercular activities [10], antiviral [11], anti-Parkinson disease [12] and anticancer agents [11,13].

**Figure 1:**
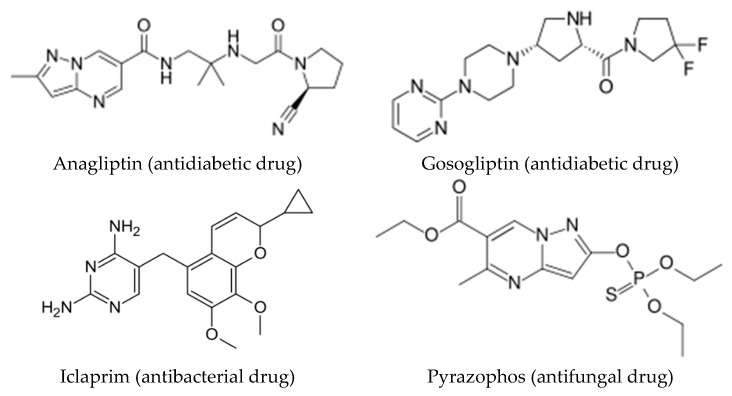
Available marketed antidiabetic and antibacterial drugs containing pyrimidine derivatives.

Several commercial antidiabetic and antimicrobial drugs containing pyrimidine derivatives have been synthesized, as shown in Figure 1. Based on these promising findings and our interest in the synthesis of biological active compounds and screening for their pharmacological evaluation [14,15,16,17] we describe herein a facile synthesis of some pyrimidine derivative compounds prepared with the aim of exploring their antidiabetic, antimicrobial activities and histopathological effects. These thiazolopyrimidine derivatives can be viewed as possible sources of new candidates for further studies. Molecular modeling can further contribute to a better understanding of the bioactivity mechanism of these pyrimidine derivatives. The molecular modeling study referred to is an in silico effort to explain potential mechanisms of action for the novel active candidates, through the identification of, and docking into specific target proteins.

## 2. Results and Discussion

### 2.1. Chemistry

**Scheme 1:**
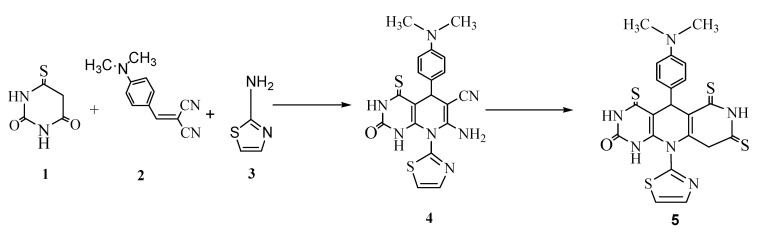
Synthesis of thiazolopyrimidine derivatives **4** and **5**.

The reaction sequences employed for the synthesis of the new compounds **4** and **5** are illustrated in Scheme 1. The compounds were synthesized referring to [18].

Prompted by the biological and medicinal activities of pyrimidines, some pyrimidine derivatives were synthesized using a one pot reaction system. The condensation reaction of 2-thioxodihydropyrimidine-4,6(1*H*,5*H*)-dione(**1**),2-(4-dimethylaminobenzylidene)malononitrile (**2**) and 2-aminothaizole (**3**) in ethanol in the presence of sodium benzoate as a non-toxic, non-flammable, clean, green, and inexpensive compound, afforded the corresponding amino-5-(4-(dimethylamino)phenyl)-hexahydro-2-oxo-8-(thiazolyl)-4-thioxopyrido[2,3-d]pyrimidinederivative **4.**

The structure of compound **4** was assigned by its spectral data (IR, ^1^H-NMR, and mass spectra). The IR spectrum of the prepared compound **4** showed absorption bands in the regions of 1170 cm^−1^ for (C=S), 1699 cm^−1^ for C=O, 2215 cm^−1^ for CN and 3150 cm^−1^ for NH_2_, in addition to a MS peak at *m/z* 422 (M^+^). The ^1^H-NMR spectrum (500 MHz, DMSO-d6, δ ppm) showed signals at 9.20 and 9.60 ppm corresponding to the two NH protons.

The condensation reaction of compound **4** with carbon disulfide CS_2_ in ethanolic solution of KOH gave the corresponding 5-(4-(10-(thiazolyl)-4,6,8-trithioxopyrimido[4,5-b][1,6]naphthyridin-2(1*H*,5*H*,10*H*)-one derivative **5.**

The structure of compound **5** was characterized by its spectral data. The IR spectrum of the product **5** displayed a strong band at 1690 cm^−1^ indicating the presence of a C=O group. Other absorption bands appeared at 3360–3375 cm^−1^ for (3NH), 1160–1175cm^−1^ for (3C=S) and the MS gave a base peak at *m/z* 497(M^+^).

### 2.2. Pharmacology

#### Antidiabetic Study

Rats were treated with compounds **4, 5** and glimepiride for ten consecutive days after which some of their biological parameters were determined (serum glucose, total antioxidant capacity (TAC) and α-amylase levels, see Table 1. Intraperitoneal (IP) injecttion of a single dose of 45 mg/kg body weight of freshly prepared streptozotocin (STZ), dissolved in citrate buffer with pH value of 4.5. A significant increase in serum glucose levels in rats of the positive control group (by 242.8%) was observed as compared to the negative control group. A significant reduction in serum glucose levels was shown in rat groups treated with glimepiride (0.1 mg/kg), compounds **4** (0.004 mg/kg) and **5** (0.006 mg/kg) by 236, 233.5 and 250.47%, respectively, when compared to positive control group. Regarding the TAC capacity, the positive control group and the groups treated with compounds **4** (0.004 mg/kg) and **5** (0.006 mg/kg) showed a significant decrease in TAC capacity compared to the negative control group by 6.25, 5.44 and 4.03%, respectively. The group treated with glimepiride (0.1 mg/kg) showed a significant increase in total antioxidant capacity compared to the negative control group and to the positive control group, by 12.21% and 17.69% respectively. A significant elevation in the activity of α-amylase by 2 fold was found in the positive control rats compared to the negative control group while the glimepiride and compounds **4** (0.004 mg/kg) and **5** (0.006 mg/kg) groups showed an inhibition of α-amylase by 55.82%, 62.94% and 75.29% compared to the positive control group.

With regard to the effect of treatment with compounds **4** and **5** and glimepiride on the serum levels of total cholesterol (TC) and high density lipoprotein (HDL-C) (Table 2), the animals treated with STZ showed a negligible increase in cholesterol levels and a significant decrease in HDL-C levels after 10 days by 47.85% compared to negative control animals, while treatment with compounds **4** and **5** resulted in a negligible increase in cholesterol levels when compared to the negative control group and a significant increase in HDL-C levels after 10 days (by 60.77% and 59.19%, respectively)when compared to the positive control group. On the other hand glimepiride produced a negligible decrease in cholesterol level by 0.5% and caused a significant increase in HDL-C levels after 10 days (by 41.27%) when compared to the positive control group.

The injection of STZ caused a significant increase in the blood glucose level in the serum of rats due to the destruction of β cells from Langerhans islets, as shown in a previous study [19]. It also resulted in a significant decrease in total antioxidant capacity and high density lipoprotein cholesterol. In addition, the histopathological study showed degenerated hypocellular atrophic renal glomeruli [20]. The hepatic tissue examination by histopathology examination revealed a widened central vein with perivascular fibrosis and aggregates of inflammatory cells in the rats that attributed the occurrence of liver damage in connection with diabetes to genetic abnormalities which could be predisposing factors for diabetes rather than hyperglycemia [21]. STZ injection elevated the level of α-amylase, which is responsible for consumption of large amounts of carbohydrates. The amylase enzyme catalyzes the breakdown of starch and complex carbohydrates to yield high levels of glucose and maltose [22].

Treatment of diabetic rats for ten successive days with glimepiride (0.1 mg/kg), a standard medicine for the treatment of diabetes, corrected most of the measured biochemical parameters and the histopathological images of the kidney and liver to be near normal (negative control group) except for HDL-C, which was less than normal, which was also the case with previous studies [23]. Regarding the treatment of diabetic rats with compounds **4** and **5**, both significantly lowered the elevated blood sugar glucose level to approximately normal levels, and they also improved the histopathologic images of both the kidneys and the liver, and they increased the HDL-C level and decreased α-amylase levels compared to the normal rats (negative control), but unfortunately they didn’t improve the TAC when compared to the untreated diabetic group (positive control).

### 2.3. Histopathology Study

**Figure 2:**
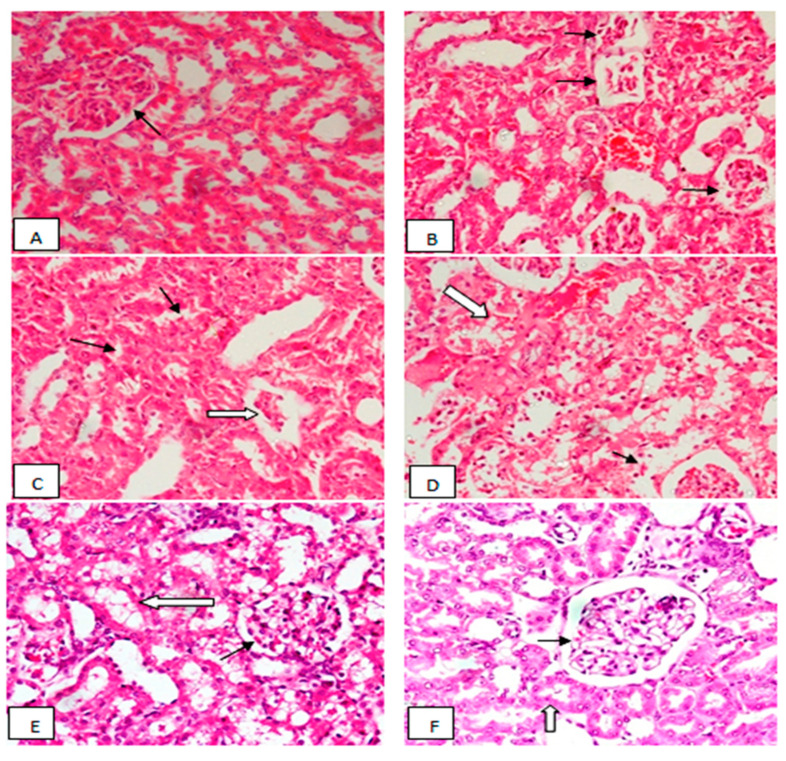
(**A**). Kidney tissue of control rat showing ordinary averagely cellular glomerulus (thin black arrow) amidst ordinary renal tubules, with low cuboidal epithelial lining and patent lumina. (**B**). Kidney tissue of diabetic untreated rat showing degenerated hypocellular glomeruli (thin black arrows) with congestion within interstitial tissue. Thick walled blood vessels were noticed. (**C**). Kidney tissue of diabetic untreated rat showing degenerated hypocellular glomerulus (white arrow) with adjacent renal tubules showing cloudy swelling (thin black arrows) within lining epithelium. (**D**). Kidney tissue of diabetic untreated rat showed renal tubules with vacuolar degeneration (white arrow) within lining epithelium. Other tubules showed atrophic lining (thin black arrow). Interstitial hemorrhages were observed. (**E**). Diabetic rat treated with reference drug showed improved glomerulus as regards cellularity (thin black arrow) with adjacent renal tubules; approximating control (white arrow). (**F**). Renal tissue of diabetic rat treated with compound (**4**) showing markedly improved glomerulus (thin black arrow) and the renal tubules (white arrow); the picture approximated control (H&E ×400).

The histopathologic examination of pancreatic tissue of rats treated with compound TBA4 showed no improvement as islets size and number were diminished compared to negative control and still showed ill-defined borders and irregular outlines, also the architecture was disrupted and constituent cells showed reduction in size and number with abundant vacuolar degeneration. However pancreatic tissue of diabetic rat treated with TBA5 showed mild improvement, with mild increase in islets number and size with disrupted architecture, constituent cells mildly increased in size and number, yet still showing abundant vacuolar degeneration and pyknosis (Figure 2).

**Figure 3:**
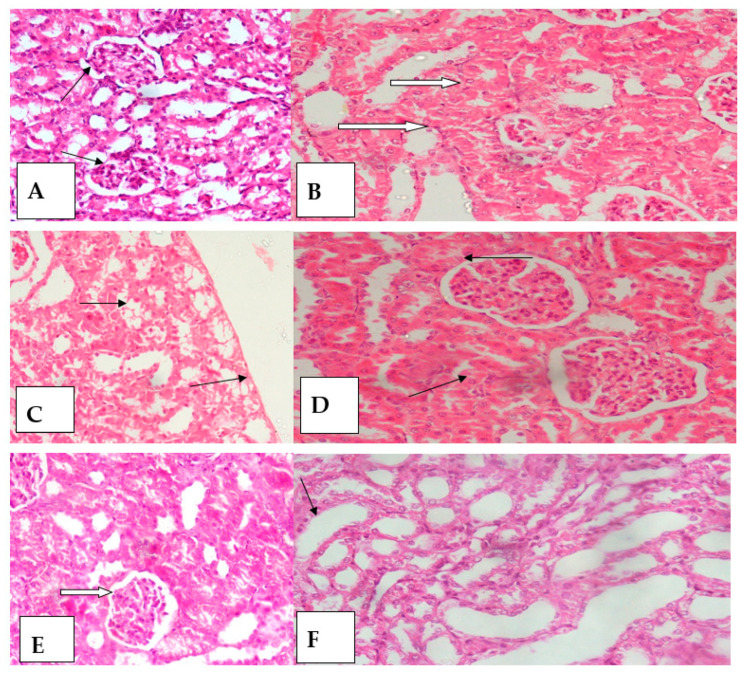
(**A**) Kidney tissue of control rat showing ordinary glomeruli (thin black arrow) with average cellularity and preserved lobular architecture. (**B**) Kidney tissue of diabetic untreated rat showing markedly degenerated, hypocellular, atrophic glomeruli (white arrows). (**C**) Kidney tissue of diabetic untreated rat showing vacuolar degeneration within epit helium lining renal tubules (thin black arrows). (**D**) Kidney tissue of diabetic rat treated with reference drug showing marked improvement within glomeruli, with restored cellularity and architecture (thin black arrows); approximating control. (**E**) Diabetic rat treated with compound **5** showing improvement within glomerular cellularity and lobulations (white arrow). (**F**) Kidney tissue of diabetic rat treated with compound **5** showed improvement within renal tubules; being lined by intact low cuboidal epithelium with patent lumina (thin black arrow). (H&E ×400).

The histopathological study found that compounds **4** and **5** showed reduced degenerative changes in liver and kidney tissue. The control rat kidney tissue generally showed cellular glomeruli surrounded by renal tubules lined with a low cuboidal epithelium (Figure 2A and Figure 3A), Diabetic untreated rats showed significantly shrunk hypocellular glomeruli. The tubules showed a lining epithelium with cloudy swelling and vacuolar degeneration. Thick-walled blood vessels were apparent (Figure 2B–D and Figure 3B,C). Kidney tissue from diabetic rats treated with each of the reference drugs, Compounds **4** and **5** showed an improvement in the glomerular cellularity and epithelium of the tubular lining (Figure 2E,F and Figure 3D–F).

**Figure 4:**
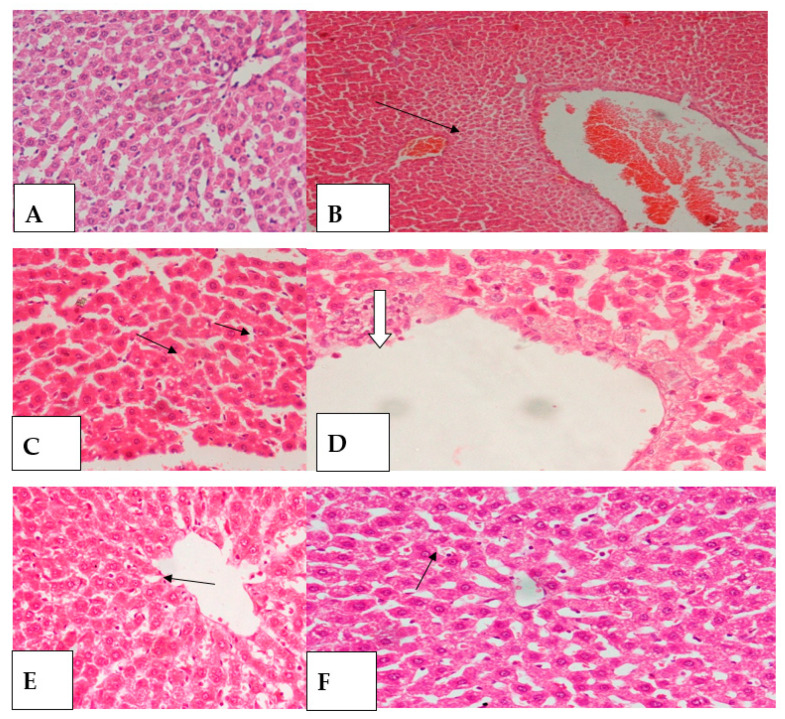
(**A**). Liver tissue of control rat showing preserved architecture with cords of linehepatocytes radiating from it with intervening sinusoids (H&E × 400). (**B**). Liver tissue of diabetic untreated rat showing marked dilatation and congestion of central vein (thin black arrow) (H&E ×100). (**C**). Liver tissue of diabetic untreated rat showing micro vesicular vacuolar degeneration (thin black arrows) within hepatocytes (H&E ×400). (**D**). Liver tissue of diabeticun treated rat showing widely dilated central vein, with perivascular fibrosis and aggregates of inflammatory cellsat periperhy (white arrow) (H&E ×400). (**E**). Liver tissue of diabetic rat treated with reference drug showing preserved architecture and ordinary hepatocytes; with residual, focal, mild micro vesicular degeneration (thin black arrow (H&E ×400). (**F**). Liver tissue of diabetic rat was treated with compound (**4**) showing marked improvement, preserved architecture and hepatocytes approximating ordinary ones; with residual, focal, very mild micro vesicular degeneration (thin black arrow) (H&E ×400).

**Figure 5:**
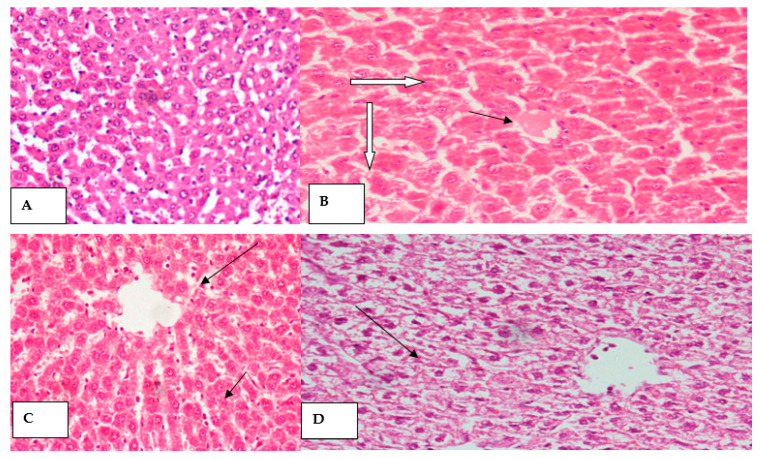
(**A**). Liver tissue of control rat showing cords of ordinary hepatocytes with sinusoids in between. (**B**). Liver tissue of diabetic untreated rat showing marked vacuolar and hydropic degeneration (white arrows) within hepatocytes with focal oedema (thin black arrow). (**C**). Liver tissue of diabetic rat treated with reference drug showing preserved architecture with ordinary hepatocytes showing minimal residual micro vesicular degeneration (thin black arrow S). (**D**). Liver tissue of diabetic rat treated with (compound **5**) showing hepatocytes with hydropic degeneration (thin black arrow). (H&E ×400).

The liver tissue of the control rats showed a conserved architecture. Ordinary polyhedral hepatocytes were seen to be arranged in cords, one cell of which was thick; radiate from a central vein (Figure 4A and Figure 5A). The liver tissue of a diabetic untreated rat showed significantly enlarged and blocked central veins. Perivascular aggregates of inflammatory cells were observed focally with perivascular fibrosis. Hepatocytes showed pronounced vacuolar, macro vesicular and degeneration together with hydropic degeneration (Figure 4B–D and Figure 5B).

Liver tissue from diabetic rats treated with each of the reference drugs, compounds **4** and **5**, showed an improvement in preserved architecture. The central veins showed no dilatation and no congestion. However, the hepatocytes still showed minimal residual micro vesicles and macro vesicles in the cytoplasm (Figure 4E,F and Figure 5C,D).

The histopathological examination results of the group treated with glimepiride were consistent with the biochemical results of the same group as the pancreatic tissue photomicrograph revealed marked improvement in both the islets and constituent cells. Islets number increased. Islet size also increased, showed regular outline and well defined borders. Islets cellularity increased. Constituent cells showed ample granular cytoplasm. Architecture and cellularity were restored. No vacuolar degeneration was noticed. No necrosis was seen. Regarding the liver tissue; the architecture was preserved, central veins showed no dilatation and no congestion, hepatocytes showed polyhedral shape with ample cytoplasm, being disposed in cords radiating from central veins, yet, hepatocytes still showed minimal, residual, micro-vesicles within the cytoplasm. As for kidney tissue, marked improvement in glomeruli and tubules was apparent, the picture approximating control.

On the other hand, the histopathology of pancreatic tissue of rats treated with compound **4** showed no improvement as islets size and number were diminished compared to negative control and still showed ill-defined borders and irregular outlines, also the architecture was disrupted and constituent cells showed reduction in size and number with abundant vacuolar. However pancreatic tissue of diabetic rats treated with compound **5** showed mild improvement, with mild increase in islet number and size with disrupted architecture, constituent mildly increased in size and number, yet still showing abundant vacuolar degeneration and pyknosis. Regarding liver tissue of groups treated with compound **4** or **5**, the histopathology revealed vacuolar degeneration, most prominent being macro-vesicular in rats treated with compound **4** and less so in rats treated with compound **5**. Renal tissue of groups treated with compound **4** and compound **5** showed prominent improvement within both glomeruli and tubules, glomeruli showed average cellularity, tubules showed patent lumina, being lined by low cuboidal epithelium, no vacuolar degeneration, no blood casts and no interstitial congestion [24,25].

### 2.4. Antimicrobial Activity

As shown in Table 3, compounds **4** and **5** exhibited antimicrobial activities against the Gram positive bacterium *Bacillus cereus* and the Gram negative bacteria *E.coli, Pseudomonas aeruginosa*, and *Salmonella typhimurium*. In addition, the tested compounds had antifungal activity against *Rhizopus oligosporus* when compared to the reference drug cephradine. Compounds **4** and **5** displayed significant activity against *Candida albicans*. It is noticed that compound **5** had higher biological activity than compound **4** under all the experimental conditions. Table 4 presents the minimum inhibition concentration (MIC) values for compound **5** against both *E.coli*, *Salmonella* and *Rhizopus oligosporus* was 5 µL.

### 2.5. Molecular Modeling Study

The aim of molecular docking is to provide a prediction of the ligand-receptor complex structure using computational methods. Docking can be accomplished by two interconnected steps: (a) sampling conformations of the ligand in the active center of the protein; (b) ranking these conformations using a scoring function. In addition, the sampling algorithms should be able to reproduce the experimental binding mode and provide the order of the scored docking poses.

In order to employ molecular docking to explain the differences of the compounds in their antimicrobial, antifungal and antidiabetic effects in vivo, we first set out to identify molecular targets for the compounds. To this effect we have employed services for protein target identification based on compounds structures in the public domain (see methods section). These “target fishing” procedures resulted in the identification of dipeptidyl peptidase IV (DPP-IV) and β-lactamase (AMPC) as a potential target for antidiabetic and antimicrobial activity respectively. However, this methodology failed to identify any targets explaining the antifungal activity of the molecules and hence a literature search was conducted to identify potential targets. Sterol 14-α-demethylase (CYP51) was selected as a ubiquitous antifungal target for our molecular docking studies.

#### 2.5.1. Antibacterial Docking Simulation

**Figure 6:**
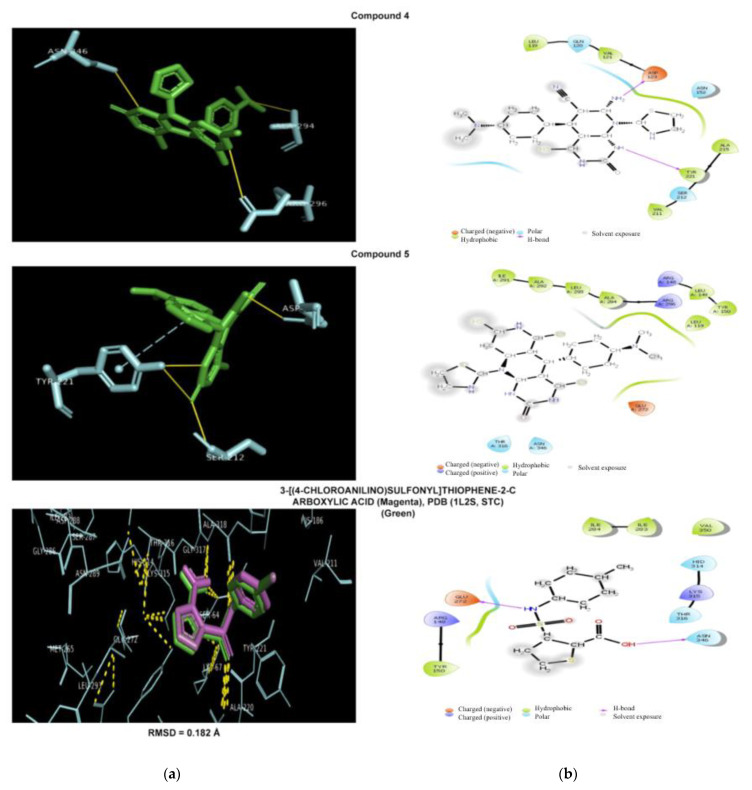
The binding states (**a**) is showing hydrogen bonds residues coloredin cyan and 2D interaction diagrams (**b**) for the compounds **4**, **5** and the native ligand (3-[(4-chloroanilino) sulfonyl] thiophene-2-carboxylic acid) in co-crystalized state ligand (green) and docked state (magenta) against β-lactamase (AMPC) from PDB (1L2S).

(3-[(4-Chloroanilino)sulfonyl] thiophene-2-carboxylic acid, active against β-lactamase (AMPC), interacts with the binding pocket via residues Ser64, Lys67, Tyr150, Asn152, Tyr221, Lys315 and Ala318 and by structural analysis the interaction complex residues Asn152, Ala318, Ser64, Tyr221 were flexibly docked. Both compounds **4** and **5** had better docking scores (Table 5) than 3-[(4-chloroanilino) sulfonyl] thiophene-2-carboxylic acid. While compound **4** showed hydrogen-bond interaction with Asp123, Ser212, and Tyr221 with π-stacking at T (Tyr221), compound **5** showed hydrogen bonding with Ala194, Arg296, and Asn346 (Figure 6, Table 6).

#### 2.5.2. Antifungal Docking Simulation

**Figure 7:**
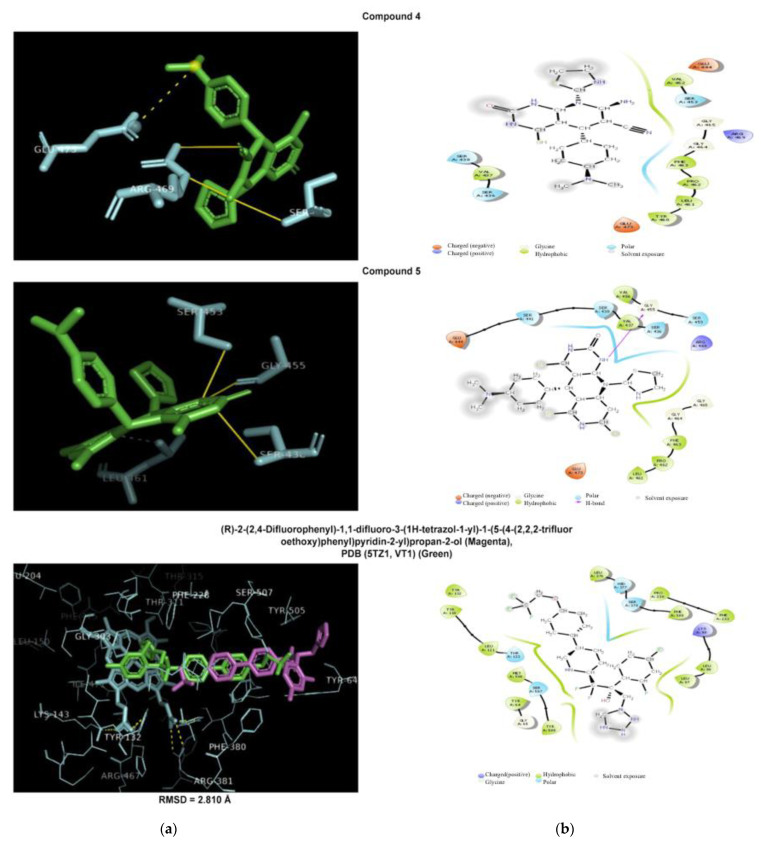
The binding states (**a**) is showing hydrogen bonds residues coloredin cyan and 2D interaction diagrams (**b**) for the compounds **4**, **5** and the candidate VT-1161 based on the native ligand (VT1) in co-crystalized state ligand (green) and docked state (magenta) against sterol 14-α-demethylase (CYP51) from *Candida albicans*, obtained from the PDB structure (5TZ1).

The binding pocket of the tetrazole-based candidate VT-1161 against sterol-14-α-demethylase (CYP51) from *Candida albicans*, which was obtained from the PDB structure (5TZ1), was further analyzed in order to reveal the characterized of the necessary interactions of the binding site. VT-1161 interacts with the following residues of its binding pocket: Tyr-64, Tyr-118, Leu-121, Thr-122, Phe-126, Ile-131, Tyr-132, Phe-228, Pro-230, Phe-233, Gly-303, Ile-304, Gly-307, Gly-308, Thr-311, Leu-376, His-377, Ser-378, Phe-380, Tyr-505, Ser-507 and Met-508 and analysis of hydrogen binding in the His-377 interaction complex was flexibly docked. Both compounds **4** and **5** have comparable docking scores of −7.3 and −7.2 kcal/mol, respectively, but were lower than the VT1 score of 10.7, probably due to the loss of the Fe-N coordination bond interaction in the activity of both compounds and lack of hydrogen bonding with His-377. While compound **4** formed hydrogen bonds with Glu444, Ser453, and Arg469 and a salt bridge on Glu473 with the tertiary amine group of compound **4** and compound **5**, they showed a hydrophobic interaction with Leu461 and hydrogen bonds with Ser438, Ser453, and Gly455 (Figure 7, Table 5 and Table 6).

#### 2.5.3. Antidiabetic Docking Simulation

**Figure 8:**
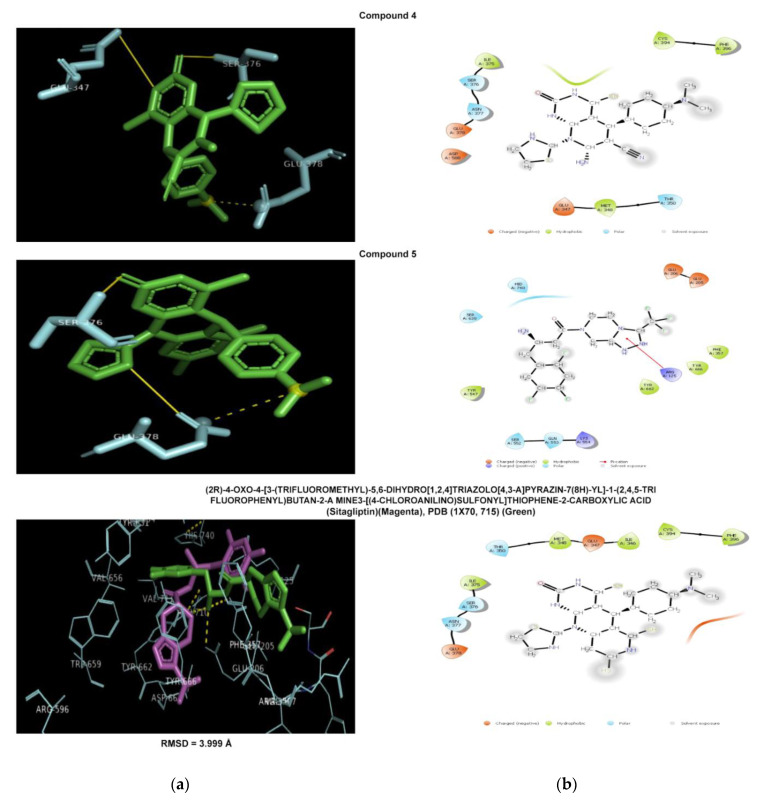
The binding states (**a**) shows hydrogen bonds residues coloredin cyan and 2D interaction diagrams (**b**) for the compounds **4** and **5** and sitagliptin in co-crystallized stateligand (green) and docked state (magenta) against human dipeptidyl peptidase IV obtained from the PDB structure (1 × 70).

Sitagliptin interacts with its DPP-IV binding pocket through three binding pockets S1, S2 and S3. S1 includes Tyr547, Ser630, Tyr631, Val656, Trp659, Tyr662, Tyr666, Asn710, Val711, and His740. S2 is the cavity approximate to Glu205, Glu206, and Tyr662 while S3 consists of Ser 209, Arg358, and Phe357. After analysis of interaction complex residues Glu205, Glu206, and Tyr662 Phe357 were forms flexible for docking purposes. Both compounds **4** and **5** achieved better docking affinities of −8.2 kcal/mol in comparison to sitagliptin of −8.0 kcal/mol (Table 5). While compound **4** formed hydrogen bonding with Glu347 and Ser376 as well as a salt bridge through its tertiary amine group at Glu378, compound **5** formed hydrogen bonds with Ser376, Glu378 and a Salt bridge at Glu378 (Figure 8, Table 5 and Table 6).

Structure-based virtual screening molecular modeling, most commonly used to predict binding modes and binding affinities, was applied to each compound **4** and **5** in the data structure and the model of the target receptor. The use of docking programs prior to experimental screening can be viewed as powerful computational filter to reduce the labor and cost of developing effective drugs as antimicrobial and antidiabetic agents. When used after the experimental screening, they can contribute to a better understanding of the bioactivity mechanisms of the tested compounds **4** and **5** to design better compounds. The current study is expected to provide useful insights into the design of antidiabetic and antimicrobial agents. This molecular docking study was also performed on synthesized compounds to support the experimental results, which agreed well with the computational results of compounds **4** and **5**.

## 3. Experimental Section

### 3.1. General Information

The precursor pyrimidine derivatives were prepared as illustrated in Scheme 1. The prepared compounds were tested for their antidiabetic and antimicrobial activities and histopathological activity. All solvents and reagents were purchased from Sigma-Aldrich (Darmstadt, Germany), and were used without any further purification. ^1^H and ^13^C nuclear magnetic resonance (NMR) spectra were recorded at the National Research Center (Cairo, Egypt) in DMSO-d_6_ on a JEOL spectrometer at 500 MHz (Tokyo, Japan). Chemical shifts are given in ppm. The melting points of solid derivatives were measured using an Electro thermal melting point apparatus were determined in open capillaries using a SMP30 digital melting point apparatus (Stuart, Staffordshire, UK). The progress of all the reactions were monitored by thin-layer chromatography (TLC) aluminum sheets pre-coated with silica gel 60F254 with a layer thickness of 0.25 mm (Merck, Darmstadt, Germany). The IR spectra (4000–400 cm^−1^) were recorded using KBr pellets in a FT/IR 300E Fourier transform infrared spectrophotometer(Jasco, USA)The mass spectra were recorded at the National Research Center (Cairo, Egypt) using a GC-MS Finnegan spectrometer (Thermo Fisher, Bedford, MA, USA) at 70 eV.

### 3.2. Synthesis of 7-Amino-5-(4-(dimethylamino)-phenyl)-1,2,4,6-hexahydro-2-oxo-8-(thiazolyl)-4-thioxopyrido [2,3-d] pyrimidine-6-carbonitrile (***4***)

A mixture of 2-thioxo-dihydropyrimidine-4,6(1*H*,5*H*)dione(**1**, 0.01 mol),2-(4-dimethylamino-benzylidene) malononitrile (**2**, 0.01 mol) and 2-aminothaizole (**3**, 0.01 mol) in absolute ethanol(20 mL) containing sodium benzoate(5% mol)was heated under reflux with stirring for 3 h at 100 °C (the progress of the reaction was monitored by TLC). After completion the reaction, the product was filtered, washed with water (50 mL), dried under vacuum and recrystallized from absolute ethanol to give the pure product **4**. Yield 86%, mp190–192 °C; IR: cm^−1^: 3340–3455 (2NH), 3150 (NH_2_), 2170 (CH-aromatic), 1699 (C=O), 1170(C=S), 2215(CN); ^1^H-NMR (500 MHz, DMSO-d_6_, δ ppm) 2.25 (3H, s, CH_3_), 2.39 (3H, s, N-CH_3_), 4.60 (1H, s, CH), 4.90 (1H, d, CH), 5.45 (1H, s, CH), 6.40 (2H,s,NH_2_), 6.90–7.20 (4H,m,Ar-H), 9.20 (1H,s,NH), 9.60((1H,s,NH); ^13^C NMR (500 MHZ), DMSO-d_6_): 25.00 (CH_3_), 40.90 (N-CH_3_), 108.00–135.60 (C-aromatic), 129.90(C=C), 148.60(C=O), 160.00(C=N), 170.80(C=S), Anal.Calc: For C_19_H_17_N_7_OS_2_(423.51); C, 53.88; H, 4.05; N, 23.15; S, 15.14; MS *m/z* (%): 422 (M^+^−1).

### 3.3. Synthesis of 5-(4-(Dimethylamino)phenyl)-3,4,6,7,8,9-hexahydro-10-(thiazolyl)-4,6,8-trithioxopyrimido [4,5-b] naphthyridine-2 (1H, 5H, 10H)-one (***5***)

A mixture from compound **4** (1 mmol) and carbon disulfide (1 mmol) in the presence of ethanolic KOH (5 mL, 10% in ethanol)was stirred at room temperature for 30 min, then heated under reflux with stirring for 5 h at 150 °C.(the progress of the reaction was monitored by TLC). After cooling, the formed solid product was extracted with ethyl acetate and washed with brine several times and then with water (3 × 20 mL), dried over anhydrous MgSO_4_ and concentrated to under vacuum. The product was purified by recrystallization from acetone to afford compound **5.** Yield 75%, mp 215–217 °C; IR: cm^−1^: 3360–3375 (3NH), 2180 (CH-aromatic), 1690 (C=O), 1160–1175 (3C=S); 1H-NMR (500 MHz, DMSO-d_6_, δ ppm), 2.68 (3H, s, CH_3_), 2.90 (3H, s, N-CH_3_), 4.30 (2H, s, CH_2_), 5.10 (1H, s,CH), 5.40 (1H, d, CH), 5.60 (1H, s, CH), 7.00–7.40 (4H, m, Ar-H), 9.20 (1H, s, NH), 9.45(1H,s,NH), 9.80 (1H,s,NH), ^13^C NMR (500 MHZ), DMSO-d_6_): 24.00 (CH_3_), 30.90 (CH_2_), 35.90 (N-CH_3_), 110.00, 130.80 (C-aromatic), 139.90(C=C), 145.90(C=C), 158.90(C=N), 170.40 (C=O), 188.60 (C=S), 195 (C=S), Anal.Calc: For C_21_H_18_N_6_OS_4_ (498.67); C, 50.58; H, 3.64; N, 16.85; S, 25.72; MS *m/z* (%):497 (M^+^−1).

### 3.4. Antidiabetic Activity Study

#### 3.4.1. Animals

Male albino Wistar rats (200 g b.w.) were obtained from animal house colony of the National Research Centre (Dokki, Cairo, Egypt).All animals were housed in stainless steel cages in a temperature controlled (23 ± 1 °C) and artificially illuminated (12 h dark/light cycle) room free from any source of chemical contamination, and were provided with a standard laboratory diet and water ad libitum. Animal procedures were performed in accordance with the Ethics Committee of the National Research Centre and followed the recommendations of the National Institutes of Health Guide for Care and Use of Laboratory Animals

#### 3.4.2. Reagents and Tested Compounds

Glimepiride, purchased from Sanofi-Aventis, Sandoz Glimepiride, Sandoz, Boucherville, QC, Canada).

Thiazolopyrimidine derivatives **4** and **5**.

#### 3.4.3. Diagnostic Kits

Kits For detection of glucose, total antioxidant capacity (TAC), α-1mylase, total cholesterol (TC) and high-density lipoprotein cholesterol (HDL-C) levels in rat serum were purchased from Biodiagnostic lab (Dokki, Giza, Egypt).

#### 3.4.4. Experimental Design

Forty male Wister rats were equally divided into five groups: the negative control group receiving 1 mL of distilled water per day for 10 consecutive days and four groups of diabetic rats in which diabetes was induced by fasting for 24 h and subsequent intraperitoneal injection of a single dose of 45 mg/kg body weight of freshly prepared streptozotocin (STZ), dissolved in citrate buffer at pH 4.5, according to [19,20,21,22,23,24,25,26] After 48 h, the blood glucose level was measured for all rats. Rats with glucose levels greater than 300 mg/dL [27]. Rats were divided into four subgroups of eight rats each as follows: the positive control group that received no treatment throughout the experiment. The rats in the reference group received glimepiride p.o. for 10 consecutive days in a dose of 0.1 mg/kg body weight [20]. The fourth and fifth groups received compound **4** p.o. in a dose of 0.004 mg/kg body weight and compound **5** p.o. in a dose of 0.006 mg/kg body weight for ten consecutive days.

#### 3.4.5. Biochemical Assays

Twenty four hours after the last dose, blood was obtained from all groups of rats after being lightly anaesthetized by puncturing retro-orbital plexus [28] using thiopental (45 mg/kg; intraperitoneal [i.p.]) [29]. The blood was allowed to flow into a clean dry centrifuge tube and left to stand 30 min before centrifugation to avoid hemolysis. Then blood samples were centrifuged for 15 min at 3000 rpm [30,31,32]. and the clear supernatant serum was separated then collected by Pasteur pipette into a dry clean tube to use for determination of serum levels of glucose, TAC, α-amylase, TC and HDL-C according to [33,34,35].

### 3.5. Histopathological Tests

At the end of the experiment; animals were sacrificed by decapitation [36]. Pancreas (splenic part), livers and kidney were dissected and extracted from sacrificed animals. Organ tissues were fixed in 10% buffered formalin, processed through ascending grades of alcohol, cleared in xylene and prepared into paraffin blocks. Serial sections 5 microns thick were prepared from each block and stained with haematoxylin and eosin for routine histopathologic study [37,38]. The sections were examined under a CX41 research microscope (Olympus, USA) at the National Research Centre Pathology Department. Slide tissue microphotography was done using an Olympus DP-12 CCD digital camera attached to the Olympus CX41 research microscope. Digital photo micrographic sections were taken at various magnifications.

### 3.6. Statistical Analysis

The means were compared using a one-way analysis of variance (ANOVA), followed by Tukey Kramer. *p* < 0.05 was considered significant. The GraphPad prism software (version 5; San Diego, CA, USA) was used to carry out the statistical analysis tests.

### 3.7. Antimicrobial Activity Assay

The tested compounds were examined for their in vitro antimicrobial activities, which are carried out against several bacterial strains. The results showed that compounds **4** and **5** tested showed antimicrobial activity ranging from moderate to good activities. They were determined using the diffusion plate method. A sterilized filter paper disc saturated with a measured quantity (25 µL) of the sample (1 mg/mL) is placed on a plate (9 cm diameter) containing a solid bacterial medium (nutrient agar) or a fungal medium (potato dextrose agar) which has been seeded with the spore suspension of the tested organism. After incubation at 37 °C for 24 h for bacteria (in the case of fungi, at 25 °C for 72 h), the diameter of the clear zone of inhibition surrounding the sample is taken as a measure of the inhibitory power of the sample against the particular tested organism (% inhibition = sample inhibition zone (cm)/plate diameter × 100). All measurements were done in DMSO as a solvent which has zero inhibition activity. The antimicrobial activity of the tested compounds were examined with the Gram positive bacteria*, Bacillus cereus, Staphylococcus aureus* ATCC 6538*, Salmonella typhimurium ATCC 25566* and the Gram negative bacteria *Escherichia coli* NRRN 3008 and *Pseudomonas aeruginosa* ATCC 10145 and the fungus *Candida albicans* EMCC105. The obtained results are compared with the reference antibiotic cephradine purchased from a local pharmacy [39,40,41,42].

### 3.8. Molecular Modeling Study

#### 3.8.1. Target Fishing Simulation

Target fishing for the synthesized compounds **4** and **5** was performed using two computational tools: SwissTargetPrediction Server [43,44,45,46] as a tool for reverse docking and determination of potential target families for a specific compound and Polypharmacology Browser (3) tool as a multi-fingerprint based prediction method that could compute possible analogs of shared fingerprints through an extensive search through 4613 chemical groups of curated molecules in ChEMBL database. The target fishing procedure was performed with the aim of providing potential targets that explain the experimental antibacterial, antifungal and antidiabetic activities of the synthesized compounds **4** and **5**.

#### 3.8.2. Antidiabetic Target Fishing Simulation

SwissTargetPrediction tool predicted DPP-IVas a potential target of the synthesized compound **4**, DPP-IV belongs to the protease family, the prediction results were further confirmed with Polypharmacology browser which predicted DPP-IV as a potential target for both compounds **4** and **5** which might help toexplainthe experimental antidiabetic data.

In order to search the potential targets for explain the antimicrobial activity of the compounds, the Polypharmacology browser prediction results were manually filtered and beta lactamase (AMPC) was found as a potential target for both compounds **4** and **5**. As these tools weren’t able to account for antifungal druggable targets, a literature search was done to select targets for the computational validation through docking simulations. Sterol 14-α-demethylase CYP51 was selected as a potential antifungal target across various fungal species (Supplementary File 1).

#### 3.8.3. Ligands and Targets Docking Simulation

The 2D representations of the two compounds **4** and **5** and the native ligands (3-[(4-chloroanilino) sulfonyl] thiophene-2-carboxylic acid from AmpC β-lactamase obtained from the PDB Structure 1L2S [47,48,49], tetrazole-based candidate VT-1161 of sterol-14-α-demethylase (CYP51) from *Candida albicans* from the PDB structure 5TZ1 [50]and sitagliptin from human dipeptidyl peptidase IV from the PDB structure [1 × 70] were obtained in MOL format and then subjected to energy minimization using Chem3D Ultra through the MM2 force field, resulting in a semi-empirical AMI method of the MOPAC module with root mean square (RMS)gradients of 0.1. The prepared structures were then converted to PDBQT format by MG L tools before docking [50].

The structure of the targets; Amp C α-Lactamase, CYP51 and DPP-IV were optimized by removing water and solvent atoms, keeping the interaction configuration in its desired state for docking purposes. The generated structures were further prepared for docking by adding polar hydrogen atoms and merging non-polar hydrogen atoms using MGL tools. Active binding residues of targets were taken from their published literature, in which the docking grid box was made and on the active sites of 3-[(4-chloroanilino) sulfonyl] thiophene-2-carboxylic acid on β-lactamase-PDB (1L2S) was centered.

The VT-1161 of sterol-14-α-demethylase, (CYP51) -PDB (5TZ1) or sitagliptin on DPP-IV-PDB (1 × 70), respectively.

#### 3.8.4. Simulations Using AutoDockvina

Docking simulations were designed based on the flexibility of the target site. Therefore, the definition of flexible binding residues was carried out using AutoDock tools to select flexible residues that were retrieved from a published literature on the aforementioned goals. Vina’s standard scoring function has been implemented. The 2D diagrams of the ligand interaction after docking were examined with the Protein Ligand Interaction Profiler (PLIP) (4) and the free license visualization Maestro 2015–1 (version 4), while the docking poses were visualized with PyMOL v1.1 (5). The binding positions of the docked complexes were analyzed and the amino acid residues 4 Å apart were regarded as binding partners of the ligands. The interaction diagrams, which represent the docked complexes, were created with PyMOL v1.1eval. Docking simulations were carried out with AutoDock 4.2.3 and AutoDockvina 1.1.2 with MGL-Tools 1.5.6 [51].

## 4. Conclusions

Molecular modeling is an important tool in computational drug design and is often adapted to predict the binding affinities of protein-ligand complexes, known as binding affinity prediction (BAP). The molecular modeling study revealed that compound **4** and **5** had better docking scores and hydrogen bonding compared to sitagliptin. Compound **5** achieved a better docking affinity (−8.2 kcal/mol) in comparison to sitagliptin (−8.0 kcal/mol). The prepared pyrimidine derivatives exhibited a broad range of biological activities, including antidiabetic and antimicrobial activities. In the current work, we continue our research on bioactive compounds such as pyrimidine derivatives. We have demonstrated a good efficient method for the synthesis of pyrimidine derivatives with good yield using the cheap and readily available non-toxic organocatalyst sodium benzoate. The advantage of this procedure is its simplicity, clean reaction, easy preparation and handling of the catalyst, safe, and environmental friendly reaction conditions. In the present study compounds **4** and **5** showed results near to those of glimepiride with regards to hypoglycaemic and anti-hyperlipdaemic effects. Moreover the pancreatic, liver and especially renal tissue of diabetic rats treated with compound **5** showed a similar improvement as that observed with glimepiride. These results suggest that compound **5** could be a promising hypoglycaemic agent with nephroprotective effects in diabetic individuals. The pharmacologic results of compounds **4** and **5** were complemented by a histopathological study. This study shows that compound **4** and **5** has a significant activity as an antidiabetic and antimicrobial agent, and that it has potential to be developed as a drug for selective inhibition e of α-amylase.

## Figures and Tables

**Table 1:** Effects of compounds **4** and **5** on glucose level, total antioxidant capacity and alpha amylase level in serum of STZ diabetic rats.

Groups	Glucose Level (mg/dL)	Total Antioxidant Capacity (mM/L)	α-Amylase Level (U/L)
**Negative control**	119.1 ± 5.83	4.96 ± 0.04	104.5 ± 1.569
**Positive control (STZ 45 mg/kg)**	408.3 ± 36.32 ^a^	4.65 ± 0.01 ^a^	317.4 ± 12.86 ^a^
**Glimepiride (0.1 mg/kg)**	121.5 ± 11.31 ^b^	5.65 ± 0.07 ^a,b^	140.2 ± 10.6 ^a,b^
**Compound 4(0.004 mg/kg)**	122.4 ± 3.2 ^b^	4.69 ± 0.004 ^a^	117.6 ± 1.51 ^a,b^
**Compound 5(0.006 mg/kg)**	116.5 ± 7.2 ^b^	4.76 ± 0.01 ^a^	78.41 ± 1.04 ^a,b^

The results are expressed as mean + SEM. The means were compared using a one-way analysis of variance (ANOVA), followed by several comparative tests by Tukey Kramer. N = 8, *p* < 0.05. ^a^ Significantly different from negative control group, ^b^ Significantly different from the positive control group.

**Table 2:** Effects of compounds **4** and **5** on the serum lipid profile of STZ diabetic rats.

Groups	Total Cholesterol (TC) Level (mg/dL)	High Density Lipoprotein Cholesterol (HDL-C) Level (mg/dL)
**Negative control**	238.7 ± 3.92	385 ± 4.35
**Positive control (STZ 45 mg/kg)**	241.26 ± 7.97	200.75 ± 5.5 ^a^
**Glimepiride (0.1 mg/kg)**	237.47 ± 2.56	341.83 ± 0.34 ^a,b^
**Compound 4(0.004 mg/kg)**	244.6 ± 3.87	511.78 ± 24.21 ^a,b^
**Compound 5 (0.006 mg/kg)**	244.27 ± 3.03	491.98 ± 17.37 ^a,b^

The results are expressed as mean + SEM. The means were compared using a one-way analysis of variance (ANOVA), followed by several comparative tests by Tukey Kramer. N = 8, *p* < 0.05. ^a^ Significant different from the negative control group, ^b^ Significantly different from the positive control group.

**Table 3:** The anti-bacterial and antifungal activities of the tested compounds **4** and **5.**

Inhibition Zone Diameter mm/mg				
Microorganism	Gram Strain	Compound 4	Compound 5	Reference Antibiotic
*Bacillus cereus*	positive	10	13	30
*Escherichia coli*	negative	8	10	15
*Pseudomonas aeruginosa*	negative	10	12	20
*Staphylococcus aureus*	positive	10	10	30
*Salmonella typhimurium*	negative	9	10	15
*Candida albicans*	yeast	19	25	35
*Rhizopus oligosporus*	fungus	12	15	25
*Mucormiehei*	fungus	10	10	28
*Aspergillus niger*	fungus	10	10	25

**Table 4:** Minimal Inhibitory Concentration (MIC) for the highest clear zones of the compound **5** against *E.coli, Salmonella,* and *Aspergillus niger.*

Microorganism	Clear Zone of Compound 5 Concentration (µL)			
	5 µL	10 µL	15 µL	20 µL
*E.coli*	10	10	10	10
*Salmonella typhimurium*	10	10	10	10
*Aspergillus niger*	10	15	17	15

**Table 5:** AutodockVina’s flexible docking scores for docking runs of synthesized compounds **4** and **5** and native against each of the three targets AMPC, CYP51 and DPP-IV.

Compound	AMPC	CYP51	DPP-IV
4	−7.7	−7.3	−8.2
5	−7.2	−7.2	−8.2
Native ligand	−6.5 (3-[(4-chloroanilino)sulfonyl] thiophene-2-carboxylic acid)	−10.7 (VT-1161)	−8.0 (Sitagliptin)

**Table 6:** The docking poses analysis of interaction complexes using PLIP of synthesized compounds **4** and **5** as well as native ligands against each of the three targets AMPC, CYP51 and DPP-IV.

Compound	AMPC	CYP51	DPP-IV
**4**	Hydrogen bonds (Asp123, Ser212, Tyr221) π-Stacking T(Tyr221)	Hydrogen bonds (Glu444, Ser453, Arg469) Salt bridge (Glu473) with tertamine group	Hydrogen bonds (Glu347, Ser376) Salt bridge with tertamine group (Glu378)
**5**	Hydrogen bonds (Ala194, Arg296, Asn346)	Hydrophobic (Leu461), Hydrogen bonds (Ser438), Ser453, Gly455	Hydrogen bonds (Ser376, Glu378) Salt bridge (Glu378)
**Native ligand**	[3-[(4-chloroanilino)sulfonyl]thiophene-2-carboxylic acid] Hydrophobic (Tyr221), Hydrogen bonds (Ser64, Lys76, Asn152, Ala318), Water bridge (Asn346)	Composite ligand consists of Heme and (VT-1161) Hydrophobic interaction (Tyr118, Phe126, Ile304, Thr311, Pro375, Leu376, Phe380, Phe463, Ile471, Phe475, Ala476),Hydrogen bonds (Tyr118, tyr132,His377, His468) Halogen bond (Tyr132) Salt bridges (between carboxylate group and Lys143, Arg381, His468) Heme group forms metal complex (Cys470)	(Sitagliptin) Hydrophobic (Val711),Hydrogen bonds (Glu205,Glu206,Tyr662), Water bridge (Ser209), π-Stacking P(Phe357), P(Tyr662), T(Tyr666) Halogen bond (Ser209)

## Data Availability

Not applicable.

## Supplementary Materials

The following are available online, File-1, Target Fishing Results using Polypharmacology browser Tool.

## Sample Availability

Samples of the compounds are available from the authors.
