# Supplementary material for: Promising Antidiabetic and Antimicrobial Agents Based on Fused Pyrimidine Derivatives: Molecular Modeling and Biological Evaluation with Histopathological Effect
Source: Molecules. 2021 Apr 19;26(8):2370. doi: 10.3390/molecules26082370 (PMC8072832; doi:10.3390/molecules26082370)
Supplement: Supplementary file 1 [file molecules-26-02370-s001.zip › molecules-1102920-supplementary-1.pdf]

Supplementary File-1

Target Fishing Results using Polypharmacology browser Tool

These targets have been predicted using the method described in:

Awale, M, Reymond, J- L (2017). The polypharmacology browser: a web- based multi-fingerprint target prediction tool using ChEMBL bioactivity data. J Chem 9: 11.

-

A. Ranked Target Prediction results for Compound 4

Target Name	CHEMBL Target ID	Target Symbol
Plasmodium_falciparum	CHEMBL364	ORGANISM_NOGN
Guanine_nucleotide-binding_protein_G(s),_subunit_alpha	CHEMBL4377	GNAS
Mothers_against_decapentaplegic_homolog_3	CHEMBL1293258	SMAD3
Histone-lysine_N-methyltransferase,_H3_lysine-9_specific_3	CHEMBL6032	EHMT2
Anthrax_lethal_factor	CHEMBL4372	LEF
Beta-lactamase_AmpC	CHEMBL2026	AMPC
Geminin	CHEMBL1293278	GMNN
SNB-75	CHEMBL614451	CELL-LINE_NOGN
Malme-3M	CHEMBL614021	CELL-LINE_NOGN

MOLT-4	CHEMBL614177	CELL-LINE_NOGN
Nuclear_factor_erythroid_2-related_factor_2	CHEMBL1075094	NFE2L2
Thioredoxin_glutathione_reductase	CHEMBL6110	TGR
HOP-92	CHEMBL613889	CELL-LINE_NOGN
SNB-19	CHEMBL614164	CELL-LINE_NOGN
Endoplasmic_reticulum-associated_amyloid_beta-peptide-binding_protein	CHEMBL4159	HSD17B10
HepG2	CHEMBL395	CELL-LINE_NOGN
Arachidonate_15-lipoxygenase,_type_II	CHEMBL2457	ALOX15B
Putative_fructose-1,6-bisphosphate_aldolase	CHEMBL1293234	ALD
Nuclear_receptor_ROR-gamma	CHEMBL1293231	RORC
K562	CHEMBL385	CELL-LINE_NOGN
Glucagon-like_peptide_1_receptor	CHEMBL1784	GLP1R
Arachidonate_15-lipoxygenase	CHEMBL2903	ALOX15
Inositol_monophosphatase_1	CHEMBL1293238	IMPA1
Ataxin-2	CHEMBL1795085	ATXN2
SR	CHEMBL614300	CELL-LINE_NOGN
Androgen_Receptor	CHEMBL3072	AR

SK-OV-3	CHEMBL614925	CELL-LINE_NOGN
Protein-tyrosine_phosphatase_1B	CHEMBL335	PTPN1
DNA_polymerase_iota	CHEMBL5391	POLI
Thioredoxin_reductase_1,_cytoplasmic	CHEMBL6035	TXNRD1
11-beta-hydroxysteroid_dehydrogenase_1	CHEMBL4235	HSD11B1
Thyroid_stimulating_hormone_receptor	CHEMBL1963	TSHR
Cellular_tumor_antigen_p53	CHEMBL4096	TP53
Neuropeptide_Y_receptor_type_5	CHEMBL4561	NPY5R
ATP-dependent_Clp_protease_proteolytic_subunit	CHEMBL2146309	CLPP
Rattus_norvegicus	CHEMBL376	ORGANISM_NOGN
HCT-116	CHEMBL394	CELL-LINE_NOGN
Aldehyde_dehydrogenase_1A1	CHEMBL3577	ALDH1A1
Cannabinoid_CB2_receptor	CHEMBL253	CNR2
Mus_musculus	CHEMBL375	ORGANISM_NOGN
Glycoprotein_hormones_alpha_chain	CHEMBL2146305	CGA
Nonstructural_protein_1	CHEMBL1293303	NS1
Huntingtin	CHEMBL5514	HTT

Dipeptidyl_peptidase_IV	CHEMBL3813	DPP4
CH1	CHEMBL614455	CELL-LINE_NOGN
Hepatocyte_nuclear_factor_4-alpha	CHEMBL5398	HNF4A
Perilipin-1	CHEMBL1741164	PLIN1
Glycosyltransferase-like_protein_LARGE1	CHEMBL2146300	LARGE
LoVo	CHEMBL614721	CELL-LINE_NOGN
Dipeptidyl_peptidase_IV	CHEMBL4653	DPP4
Sphingomyelin_phosphodiesterase	CHEMBL2760	SMPD1
Protein_RecA	CHEMBL1741171	RECA
A2780	CHEMBL614004	CELL-LINE_NOGN
Dipeptidyl_peptidase_VIII	CHEMBL4657	DPP8
Chromobox_protein_homolog_1	CHEMBL1741193	CBX1
HT-29	CHEMBL384	CELL-LINE_NOGN
Nuclear_receptor_ROR-gamma	CHEMBL1741186	RORC
Tumor_susceptibility_gene_101_protein	CHEMBL6157	TSG101
HMG-CoA_reductase	CHEMBL402	HMGCR
Arachidonate_5-lipoxygenase	CHEMBL312	ALOX5

Glutaminase_kidney_isoform,_mitochondrial	CHEMBL2146302	GLS
Acyl_coenzyme_A:cholesterol_acyltransferase	CHEMBL2623	NA
Neuronal_acetylcholine_receptor;_alpha4/beta2	CHEMBL1907596	CHRNA4_CHRNB2
C-C_chemokine_receptor_type_5	CHEMBL274	CCR5
Neuronal_acetylcholine_receptor_protein_alpha-7_subunit	CHEMBL4980	CHRNA7
DNA_dC->dU-editing_enzyme_APOBEC-3G	CHEMBL1741217	APOBEC3G
Glucocorticoid_receptor	CHEMBL2034	NR3C1
Lysine-specific_demethylase_4A	CHEMBL5896	KDM4A
Isocitrate_dehydrogenase_[NADP]_cytoplasmic	CHEMBL2007625	IDH1
DNA_dC->dU-editing_enzyme_APOBEC-3F	CHEMBL2007626	APOBEC3F
6-phospho-1-fructokinase	CHEMBL5686	PFK
B16	CHEMBL381	CELL-LINE_NOGN
SK-MEL-28	CHEMBL614919	CELL-LINE_NOGN
SN12C	CHEMBL614054	CELL-LINE_NOGN
RXF_393	CHEMBL614886	CELL-LINE_NOGN
786-0	CHEMBL613102	CELL-LINE_NOGN
MDA-MB-435	CHEMBL614697	CELL-LINE_NOGN

ACHN	CHEMBL614519	CELL-LINE_NOGN
HeLa	CHEMBL399	CELL-LINE_NOGN
PC-3	CHEMBL390	CELL-LINE_NOGN
Matrix_metalloproteinase_13	CHEMBL280	MMP13
P2X_purinoceptor_7	CHEMBL4805	P2RX7
Neuropeptide_S_receptor	CHEMBL5162	NPSR1
MCF7	CHEMBL387	CELL-LINE_NOGN
Saccharomyces_cerevisiae	CHEMBL361	ORGANISM_NOGN
DLD-1	CHEMBL614285	CELL-LINE_NOGN
T47D	CHEMBL614361	CELL-LINE_NOGN
Serine-protein_kinase_ATM	CHEMBL3797	ATM
Ras-related_protein_Rab-9A	CHEMBL1293294	RAB9A
Vitamin_D_receptor	CHEMBL1977	VDR
Lysosomal_alpha-glucosidase	CHEMBL2608	GAA
Alpha-synuclein	CHEMBL6152	SNCA
Beta-glucocerebrosidase	CHEMBL2179	GBA

-

B. Ranked Target Prediction results for Compound 5

MCF7	CHEMBL387	CELL-LINE_NOGN
Plasmodium_falciparum	CHEMBL364	ORGANISM_NOGN
PC-3	CHEMBL390	CELL-LINE_NOGN
HCT-116	CHEMBL394	CELL-LINE_NOGN
Prelamin-A/C	CHEMBL1293235	LMNA
OVCAR-4	CHEMBL614051	CELL-LINE_NOGN
Glycoprotein_hormones_alpha_chain	CHEMBL2146305	CGA
HeLa	CHEMBL399	CELL-LINE_NOGN
Glucagon-like_peptide_1_receptor	CHEMBL1784	GLP1R
Jurkat	CHEMBL397	CELL-LINE_NOGN
A549	CHEMBL392	CELL-LINE_NOGN
Glutaminase_kidney_isoform,_mitochondrial	CHEMBL2146302	GLS
Geminin	CHEMBL1293278	GMNN
Ataxin-2	CHEMBL1795085	ATXN2
Kinesin-like_protein_1	CHEMBL4581	KIF11

Malme-3M	CHEMBL614021	CELL-LINE_NOGN
MDA-MB-435	CHEMBL614697	CELL-LINE_NOGN
Guanine_nucleotide-binding_protein_G(s),_subunit_alpha	CHEMBL4377	GNAS
MOLT-4	CHEMBL614177	CELL-LINE_NOGN
Isocitrate_dehydrogenase_[NADP]_cytoplasmic	CHEMBL2007625	IDH1
Trypanosoma_brucei_brucei	CHEMBL612851	ORGANISM_NOGN
Glyceraldehyde-3-phosphate_dehydrogenase_liver	CHEMBL2284	GAPDH
Perforin-1	CHEMBL5480	PRF1
Staphylococcus_aureus	CHEMBL352	ORGANISM_NOGN
HepG2	CHEMBL395	CELL-LINE_NOGN
SK-MEL	CHEMBL614914	CELL-LINE_NOGN
Beta-lactamase_AmpC	CHEMBL2026	AMPC
SK-MEL-28	CHEMBL614919	CELL-LINE_NOGN
DNA_dC->dU-editing_enzyme_APOBEC-3F	CHEMBL2007626	APOBEC3F
SK-OV-3	CHEMBL614925	CELL-LINE_NOGN
MDA-MB-231	CHEMBL400	CELL-LINE_NOGN
SN12C	CHEMBL614054	CELL-LINE_NOGN

ACHN	CHEMBL614519	CELL-LINE_NOGN
786-0	CHEMBL613102	CELL-LINE_NOGN
RXF_393	CHEMBL614886	CELL-LINE_NOGN
SR	CHEMBL614300	CELL-LINE_NOGN
UACC-62	CHEMBL614610	CELL-LINE_NOGN
Chromobox_protein_homolog_1	CHEMBL1741193	CBX1
LoVo	CHEMBL614721	CELL-LINE_NOGN
Histone-lysine_N-methyltransferase,_H3_lysine-9_specific_3	CHEMBL6032	EHMT2
Serine/threonine-protein_kinase_mTOR	CHEMBL2842	MTOR
HOP-92	CHEMBL613889	CELL-LINE_NOGN
Serine/threonine-protein_kinase_Aurora-A	CHEMBL4722	AURKA
Aldehyde_dehydrogenase_1A1	CHEMBL3577	ALDH1A1
Nuclear_receptor_ROR-gamma	CHEMBL1293231	RORC
Endothelin_receptor_ET-B	CHEMBL1785	EDNRB
Endothelin_receptor_ET-A	CHEMBL252	EDNRA
Leishmania_infantum	CHEMBL612848	ORGANISM_NOGN
Homeodomain-interacting_protein_kinase_1	CHEMBL5427	HIPK1

Receptor_protein-tyrosine_kinase_erbB-2	CHEMBL1824	ERBB2
Phosphatidylinositol-4-phosphate_3-kinase_C2_domain-containing_beta_polypeptide	CHEMBL5554	PIK3C2B
AGS	CHEMBL613860	CELL-LINE_NOGN
Staphylococcus_epidermidis	CHEMBL353	ORGANISM_NOGN
Epidermal_growth_factor_receptor_erbB1	CHEMBL203	EGFR
Ephrin_type-A_receptor_4	CHEMBL3988	EPHA4
Trypanosoma_brucei_rhodesiense	CHEMBL612348	ORGANISM_NOGN
Protein_kinase_C_(PKC)	CHEMBL2094266	PRKCB_PRKCD_PRKCH_PRKCQ_PRKCE_PRKCA_PRKCG_PRKCZ
Mycobacterium_tuberculosis_H37Rv	CHEMBL2111188	ORGANISM_NOGN
Platelet-derived_growth_factor_receptor_beta	CHEMBL1913	PDGFRB
Protein_kinase_C_gamma	CHEMBL3604	PRKCG
U-937	CHEMBL612794	CELL-LINE_NOGN
Bacillus_subtilis	CHEMBL359	ORGANISM_NOGN
Transcriptional_regulator_ERG	CHEMBL1293191	ERG
Trypanosoma_cruzi	CHEMBL368	ORGANISM_NOGN
Alkaline_phosphatase,_tissue-nonspecific_isozyme	CHEMBL5979	ALPL

Serine/threonine-protein_phosphatase	CHEMBL1293265	PPP5C
Fibroblast_growth_factor_receptor_1	CHEMBL3650	FGFR1
MAP_kinase_p38_delta	CHEMBL2939	MAPK13
Vascular_endothelial_growth_factor_receptor_2	CHEMBL279	KDR
THP-1	CHEMBL614245	CELL-LINE_NOGN
Histone-lysine_N-methyltransferase_MLL	CHEMBL1293299	KMT2A
Glutamate_[NMDA]_receptor_subunit_epsilon_2	CHEMBL311	GRIN2B
Polypyrimidine_tract-binding_protein_1	CHEMBL1293230	PTBP1
DNA-(apurinic_or_apyrimidinic_site)_lyase	CHEMBL5619	APEX1
Nuclear_receptor_ROR-gamma	CHEMBL1741186	RORC
Protein_kinase_C_beta	CHEMBL3045	PRKCB
Arachidonate_5-lipoxygenase	CHEMBL215	ALOX5
Endoplasmic_reticulum-associated_amyloid_beta-peptide-binding_protein	CHEMBL4159	HSD17B10
Pyruvate_kinase	CHEMBL5149	PYK
Thyroid_stimulating_hormone_receptor	CHEMBL1963	TSHR
Protein_kinase_C_epsilon	CHEMBL3582	PRKCE
HEK293	CHEMBL614818	CELL-LINE_NOGN

Neuronal_acetylcholine_receptor_protein_alpha-7_subunit	CHEMBL4980	CHRNA7
Microtubule-associated_protein_tau	CHEMBL1293224	MAPT
6-phospho-1-fructokinase	CHEMBL5686	PFK
Lysine-specific_demethylase_4D-like	CHEMBL1293226	KDM4E
Tyrosyl-DNA_phosphodiesterase_1	CHEMBL1075138	TDP1
Protein_kinase_C_(PKC)	CHEMBL2093867	PRKCA_PRKCB_PRKCZ_PRKD1_PRKCD_PRKCH_PRKCQ_PRKCI_PRKCE_PRKD3_PRKCG
Lethal(3)malignant_brain_tumor-like_protein_1	CHEMBL1287622	L3MBTL1
Protein_kinase_C_alpha_	CHEMBL299	PRKCA
Poly_[ADP-ribose]_polymerase-1	CHEMBL3105	PARP1
Induced_myeloid_leukemia_cell_differentiation_protein_Mcl-1	CHEMBL4361	MCL1
Dipeptidyl_peptidase_IV	CHEMBL284	DPP4
Bloom_syndrome_protein	CHEMBL1293237	BLM
HT-29	CHEMBL384	CELL-LINE_NOGN
Glycogen_synthase_kinase-3_beta	CHEMBL262	GSK3B
K562	CHEMBL385	CELL-LINE_NOGN
Putative_uncharacterized_protein	CHEMBL2146316	TB927.8.6820

MDA-MB-468	CHEMBL614335	CELL-LINE_NOGN
Serine-protein_kinase_ATM	CHEMBL3797	ATM
Streptococcus_sp._'group_A'	CHEMBL612389	ORGANISM_NOGN
15-hydroxyprostaglandin_dehydrogenase_[NAD+]	CHEMBL1293255	HPGD
Putative_fructose-1,6-bisphosphate_aldolase	CHEMBL1293234	ALD
MDA-N	CHEMBL614078	CELL-LINE_NOGN
Acetylcholinesterase	CHEMBL4078	ACHE
OVCAR-3	CHEMBL614213	CELL-LINE_NOGN
DLD-1	CHEMBL614285	CELL-LINE_NOGN
NCI-H23	CHEMBL614997	CELL-LINE_NOGN
IGROV-1	CHEMBL613984	CELL-LINE_NOGN
UO-31	CHEMBL614388	CELL-LINE_NOGN
CCRF-CEM	CHEMBL382	CELL-LINE_NOGN
SK-MEL-5	CHEMBL614922	CELL-LINE_NOGN
DNA_polymerase_iota	CHEMBL5391	POLI
ATPase_family_AAA_domain-containing_protein_5	CHEMBL1741209	ATAD5
Inositol_monophosphatase_1	CHEMBL1293238	IMPA1

Mothers_against_decapentaplegic_homolog_3	CHEMBL1293258	SMAD3

Target Fishing Results using SwissTargetPrediction Tool

These targets have been predicted using the method described in:

Gfeller D., Michielin O. & Zoete V. Shaping the interaction landscape of bioactive molecules, Bioinformatics (2013) 29:3073-3079.

C. Ranked Target Prediction results for Compound 4

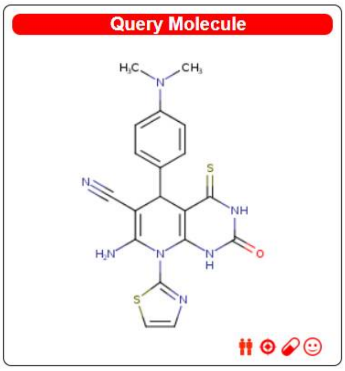

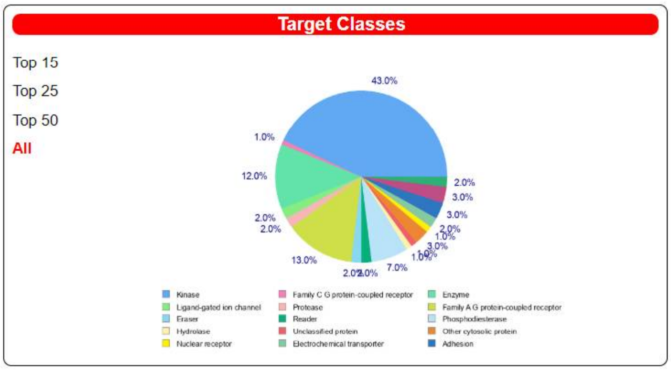

Target	Common name	Uniprot ID	ChEMBL ID	Target Class
Receptor protein-tyrosine kinase erbB-2	ERBB2	P04626	CHEMBL1824	Kinase
Metabotropic glutamate receptor 4	GRM4	Q14833	CHEMBL273	Family C G protein-coupled receptor

			6	
6-phosphofructo-2-kinase/fructose-2,6-bisphosphatase 3	PFKFB3	Q16875	CHEMBL2331053	Enzyme
Epidermal growth factor receptor erbB1	EGFR	P00533	CHEMBL203	Kinase
Tyrosine-protein kinase JAK1	JAK1	P23458	CHEMBL2835	Kinase
Cyclin-dependent kinase 2	CDK2	P24941	CHEMBL301	Kinase
Cyclin-dependent kinase 1	CDK1	P06493	CHEMBL308	Kinase
Vascular endothelial growth factor receptor 1	FLT1	P17948	CHEMBL1868	Kinase
Tyrosine-protein kinase JAK3	JAK3	P52333	CHEMBL2148	Kinase
Glycogen synthase kinase-3 beta	GSK3B	P49841	CHEMBL262	Kinase
Tyrosine-protein kinase SRC	SRC	P12931	CHEMBL267	Kinase
Vascular endothelial growth factor receptor 2	KDR	P35968	CHEMBL279	Kinase
Tyrosine-protein kinase JAK2	JAK2	O60674	CHEMBL2971	Kinase
Fibroblast growth factor receptor 1	FGFR1	P11362	CHEMBL3650	Kinase

PI3-kinase p110-alpha subunit	PIK3CA	P42336	CHEMBL4005	Enzyme
GABA A receptor alpha-2/beta-2/gamma-2	GABRA2 GABRB2 GABRG2	P47869 P47870 P18507	CHEMBL2111413	Ligand-gated ion channel
Beta-secretase 1	BACE1	P56817	CHEMBL4822	Protease
Mitogen-activated protein kinase kinase kinase 12	MAP3K12	Q12852	CHEMBL1908389	Enzyme
Serine/threonine-protein kinase RAF	RAF1	P04049	CHEMBL1906	Kinase
Adenosine A1 receptor	ADORA1	P30542	CHEMBL226	Family A G protein-coupled receptor
Adenosine A3 receptor	ADORA3	P0DMS8	CHEMBL256	Family A G protein-coupled receptor
MAP kinase p38 alpha	MAPK14	Q16539	CHEMBL260	Kinase
Histone deacetylase 1	HDAC1	Q13547	CHEMBL325	Eraser
Ephrin type-B receptor 2	EPHB2	P29323	CHEMBL3290	Kinase
Serine/threonine-protein kinase B-raf	BRAF	P15056	CHEMBL5145	Kinase
Bromodomain-containing protein 4	BRD4	O60885	CHEMBL1163125	Reader
Nicotinamide phosphoribosyltransferase	NAMPT	P43490	CHEMBL1744525	Enzyme

Phosphodiesterase 5A	PDE5A	O76074	CHEMBL1827	Phosphodiesterase
Acetylcholinesterase	ACHE	P22303	CHEMBL220	Hydrolase
Casein kinase II alpha	CSNK2A1	P68400	CHEMBL3629	Kinase
Phosphatidylinositol 3-kinase catalytic subunit type 3	PIK3C3	Q8NEB9	CHEMBL1075165	Enzyme
Tyrosine-protein kinase TYK2	TYK2	P29597	CHEMBL3553	Kinase
Stem cell growth factor receptor	KIT	P10721	CHEMBL1936	Kinase
Serine/threonine-protein kinase Aurora-B	AURKB	Q96GD4	CHEMBL2185	Kinase
c-Jun N-terminal kinase 1	MAPK8	P45983	CHEMBL2276	Kinase
Adenosine A2a receptor	ADORA2A	P29274	CHEMBL251	Family A G protein-coupled receptor
Phosphodiesterase 4B	PDE4B	Q07343	CHEMBL275	Phosphodiesterase
c-Jun N-terminal kinase 2	MAPK9	P45984	CHEMBL4179	Kinase
Serine/threonine-protein kinase Aurora-A	AURKA	O14965	CHEMBL4722	Kinase
Tyrosine-protein kinase SYK	SYK	P43405	CHEMBL259	Kinase

			9	
Phosphodiesterase 4D	PDE4D	Q08499	CHEMBL288	Phosphodiesterase
Transcription intermediary factor 1-alpha	TRIM24	O15164	CHEMBL3108638	Reader
Hepatocyte growth factor receptor	MET	P08581	CHEMBL3717	Kinase
Probable protein-cysteine N-palmitoyltransferase porcupine (by homology)	PORCN	Q9H237	CHEMBL1255163	Enzyme
EF-hand calcium-binding domain-containing protein 4B	CRACR2A	Q9BSW2	CHEMBL3638353	Unclassified protein
Phosphodiesterase 10A	PDE10A	Q9Y233	CHEMBL4409	Phosphodiesterase
Cyclin-dependent kinase 4/cyclin D1	CCND1 CDK4	P24385 P11802	CHEMBL1907601	Kinase
Melatonin receptor 1A	MTNR1A	P48039	CHEMBL1945	Family A G protein-coupled receptor
Melatonin receptor 1B	MTNR1B	P49286	CHEMBL1946	Family A G protein-coupled receptor
Cyclin-dependent kinase 2/cyclin E	CCNE2 CDK2 CCNE1	O96020 P24941 P24864	CHEMBL2094126	Other cytosolic protein
Cyclin-dependent kinase 1/cyclin B	CCNB3 CDK1 CCNB1 CCNB2	Q8WWL7 P06493 P14635 O95067	CHEMBL2094127	Other cytosolic protein

PI3-kinase p110-delta subunit	PIK3CD	O00329	CHEMBL3130	Enzyme
PI3-kinase p110-beta subunit	PIK3CB	P42338	CHEMBL3145	Enzyme
PI3-kinase p110-gamma subunit	PIK3CG	P48736	CHEMBL3267	Enzyme
Lipoxin A4 receptor	FPR2	P25090	CHEMBL4227	Family A G protein-coupled receptor
TGF-beta receptor type I	TGFBR1	P36897	CHEMBL4439	Kinase
Tyrosine-protein kinase BTK	BTK	Q06187	CHEMBL5251	Kinase
Peptidyl-prolyl cis-trans isomerase D	PPID	Q08752	CHEMBL1697657	Enzyme
Nuclear receptor ROR-gamma	RORC	P51449	CHEMBL1741186	Nuclear receptor
Insulin receptor	INSR	P06213	CHEMBL1981	Kinase
Ileal bile acid transporter	SLC10A2	Q12908	CHEMBL2778	Electrochemical transporter
Dual specificity protein kinase TTK	TTK	P33981	CHEMBL3983	Kinase
Bradykinin B1 receptor	BDKRB1	P46663	CHEMBL430	Family A G protein-coupled receptor

			8	
Lysine-specific histone demethylase 1	KDM1A	O60341	CHEMBL6136	Eraser
Pyruvate kinase isozymes M1/M2	PKM	P14618	CHEMBL1075189	Enzyme
Luteinizing hormone/Choriogonadotropin receptor	LHCGR	P22888	CHEMBL1854	Family A G protein-coupled receptor
Thyroid stimulating hormone receptor	TSHR	P16473	CHEMBL1963	Family A G protein-coupled receptor
Serotonin 2a (5-HT2a) receptor	HTR2A	P28223	CHEMBL224	Family A G protein-coupled receptor
Serotonin 2c (5-HT2c) receptor	HTR2C	P28335	CHEMBL225	Family A G protein-coupled receptor
Dual-specificity tyrosine-phosphorylation regulated kinase 1A (by homology)	DYRK1A	Q13627	CHEMBL2292	Kinase
Dual specificty protein kinase CLK1	CLK1	P49759	CHEMBL4224	Kinase
5-lipoxygenase activating protein	ALOX5AP	P20292	CHEMBL4550	Other cytosolic protein
MAP kinase-activated protein kinase 2	MAPKAPK2	P49137	CHEMBL2208	Kinase
Intercellular adhesion molecule-1	ICAM1	P05362	CHEMBL3070	Adhesion

MAP kinase-activated protein kinase 5	MAPKAPK5	Q8IW41	CHEMBL3094	Kinase
Dual specificity mitogen-activated protein kinase kinase 1	MAP2K1	Q02750	CHEMBL3587	Kinase
Vascular cell adhesion protein 1	VCAM1	P19320	CHEMBL3735	Adhesion
Selectin E	SELE	P16581	CHEMBL3890	Adhesion
MAP kinase ERK2	MAPK1	P28482	CHEMBL4040	Kinase
MAP kinase-activated protein kinase 3	MAPKAPK3	Q16644	CHEMBL4670	Kinase
Sodium/glucose cotransporter 1	SLC5A1	P13866	CHEMBL4979	Electrochemical transporter
Sodium channel protein type X alpha subunit (by homology)	SCN10A	Q9Y5Y9	CHEMBL5451	Voltage-gated ion channel
Tyrosine-protein kinase FYN	FYN	P06241	CHEMBL1841	Kinase
Tyrosine-protein kinase ABL	ABL1	P00519	CHEMBL1862	Kinase
Phosphodiesterase 2A	PDE2A	O00408	CHEMBL2652	Phosphodiesterase
Orexin receptor 2	HCRTR2	O43614	CHEMBL479	Family A G protein-coupled receptor

			2	
Vanilloid receptor	TRPV1	Q8NER1	CHEMBL4794	Voltage-gated ion channel
EZH2/SUZ12/EED/RBBP7/RBBP4	EZH2	Q15910	CHEMBL2189110	Writer
Phosphodiesterase 4A	PDE4A	P27815	CHEMBL254	Phosphodiesterase
Phosphodiesterase 7A	PDE7A	Q13946	CHEMBL3012	Phosphodiesterase
EZH2/SUZ12/EED/RBBP7/RBBP4	RBBP4 RBBP7 EED SUZ12 EZH2	Q09028 Q16576 O75530 Q15022 Q15910	CHEMBL3301388	Writer
Hexokinase type IV	GCK	P35557	CHEMBL3820	Enzyme
Cyclin-dependent kinase 5/CDK5 activator 1	CDK5R1 CDK5	Q15078 Q00535	CHEMBL1907600	Kinase
GABA-A receptor; alpha-2/beta-3/gamma-2	GABRA2 GABRB3 GABRG2	P47869 P28472 P18507	CHEMBL2094130	Ligand-gated ion channel
Cannabinoid receptor 2	CNR2	P34972	CHEMBL253	Family A G protein-coupled receptor
Chymase	CMA1	P23946	CHEMBL4068	Protease
Serine/threonine-protein kinase Chk1	CHEK1	O14757	CHEMBL4630	Kinase
Cyclin-dependent kinase 2/cyclin E1	CCNE1 CDK2	P24864 P24941	CHEMBL190	Kinase

			7605	
Insulin-like growth factor I receptor	IGF1R	P08069	CHEMBL1957	Kinase
HERG	KCNH2	Q12809	CHEMBL240	Voltage-gated ion channel

-

D. Ranked Target Prediction results for Compound 5

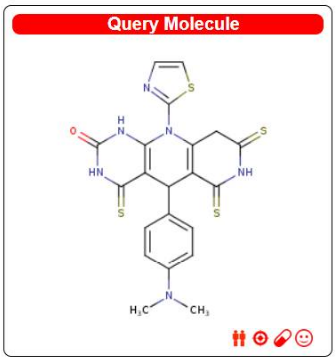

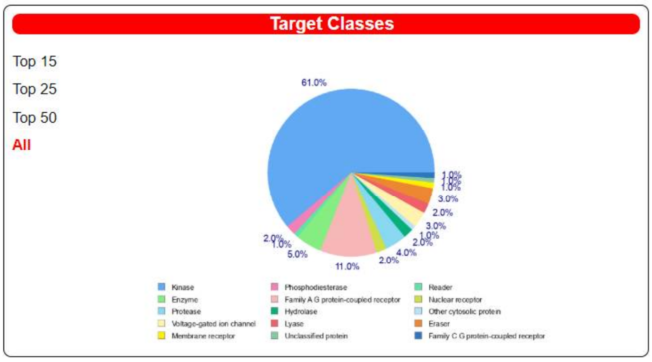

Target	Common name	Uniprot ID	ChEMBL ID	Target Class
Vascular endothelial growth factor receptor 1	FLT1	P17948	CHEMBL1868	Kinase
Vascular endothelial growth factor receptor 2	KDR	P35968	CHEMBL279	Kinase
Fibroblast growth factor receptor 1	FGFR1	P11362	CHEMBL3650	Kinase

Ribosomal protein S6 kinase alpha 3	RPS6KA3	P51812	CHEMBL2345	Kinase
Phosphodiesterase 10A (by homology)	PDE10A	Q9Y233	CHEMBL4409	Phosphodiesterase
Bromodomain-containing protein 4	BRD4	O60885	CHEMBL1163125	Reader
Pyruvate kinase isozymes M1/M2	PKM	P14618	CHEMBL1075189	Enzyme
Serine/threonine-protein kinase TBK1	TBK1	Q9UHD2	CHEMBL5408	Kinase
Melatonin receptor 1A	MTNR1A	P48039	CHEMBL1945	Family A G protein-coupled receptor
Melatonin receptor 1B	MTNR1B	P49286	CHEMBL1946	Family A G protein-coupled receptor
Serotonin 2a (5-HT2a) receptor	HTR2A	P28223	CHEMBL224	Family A G protein-coupled receptor
Serotonin 2c (5-HT2c) receptor	HTR2C	P28335	CHEMBL225	Family A G protein-coupled receptor
Glucocorticoid receptor	NR3C1	P04150	CHEMBL2034	Nuclear receptor
Beta-secretase 1	BACE1	P56817	CHEMBL4822	Protease
Cholecystokinin B receptor	CCKBR	P32239	CHEMBL298	Family A G protein-coupled receptor
Tyrosine-protein kinase TIE-2	TEK	Q02763	CHEMBL412	Kinase

			8	
Phosphodiesterase 9A	PDE 9A	O76 083	CHEM BL353 5	Phosphodiesterase
Gastrin releasing peptide receptor	GRP R	P30 550	CHEM BL495 9	Family A G protein-coupled receptor
Nuclear receptor ROR-gamma	ROR C	P51 449	CHEM BL174 1186	Nuclear receptor
Serotonin 1a (5-HT1a) receptor	HTR 1A	P08 908	CHEM BL214	Family A G protein-coupled receptor
Nerve growth factor receptor Trk-A	NTR K1	P04 629	CHEM BL281 5	Kinase
Serine/threonine-protein kinase Aurora-B	AUR KB	Q96 GD4	CHEM BL218 5	Kinase
Tyrosine-protein kinase LCK	LCK	P06 239	CHEM BL258	Kinase
Protein kinase C delta	PRK CD	Q05 655	CHEM BL299 6	Kinase
Protein kinase C theta	PRK CQ	Q04 759	CHEM BL392 0	Kinase
Serine/threonine-protein kinase Aurora-A	AUR KA	O14 965	CHEM BL472 2	Kinase
Butyrylcholinesterase	BCH E	P06 276	CHEM BL191 4	Hydrolase
Acetylcholinesterase	ACH E	P22 303	CHEM BL220	Hydrolase

11-beta-hydroxysteroid dehydrogenase 1	HSD 11B1	P28 845	CHEM BL423 5	Enzyme
Tyrosine-protein kinase SYK	SYK	P43 405	CHEM BL259 9	Kinase
5-lipoxygenase activating protein	ALO X5A P	P20 292	CHEM BL455 0	Other cytosolic protein
Cannabinoid receptor 1	CNR 1	P21 554	CHEM BL218	Family A G protein-coupled receptor
Serine/threonine-protein kinase Chk2	CHE K2	O96 017	CHEM BL252 7	Kinase
Cannabinoid receptor 2	CNR 2	P34 972	CHEM BL253	Family A G protein-coupled receptor
Voltage-gated potassium channel subunit Kv1.5	KCN A5	P22 460	CHEM BL430 6	Voltage-gated ion channel
Serine/threonine-protein kinase Chk1	CHE K1	O14 757	CHEM BL463 0	Kinase
Vanilloid receptor (by homology)	TRP V1	Q8N ER1	CHEM BL479 4	Voltage-gated ion channel
Carbonic anhydrase II	CA2	P00 918	CHEM BL205	Lyase
Carbonic anhydrase I	CA1	P00 915	CHEM BL261	Lyase
Glycogen synthase kinase-3 beta	GSK 3B	P49 841	CHEM BL262	Kinase
Tyrosine-protein kinase ITK/TSK	ITK	Q08 881	CHEM BL295 9	Kinase

Rho-associated protein kinase 2	ROCK2	O75116	CHEMBL2973	Kinase
Histone deacetylase 3	HDAC3	O15379	CHEMBL1829	Eraser
Histone deacetylase 2	HDAC2	Q92769	CHEMBL1937	Eraser
Epidermal growth factor receptor erbB1	EGFR	P00533	CHEMBL203	Kinase
Histone deacetylase 1	HDAC1	Q13547	CHEMBL325	Eraser
Tyrosine-protein kinase FYN	FYN	P06241	CHEMBL1841	Kinase
Macrophage colony stimulating factor receptor	CSF1R	P07333	CHEMBL1844	Kinase
Tyrosine-protein kinase ABL	ABL1	P00519	CHEMBL1862	Kinase
Serine/threonine-protein kinase RAF	RAF1	P04049	CHEMBL1906	Kinase
Platelet-derived growth factor receptor beta	PDGFRB	P09619	CHEMBL1913	Kinase
Stem cell growth factor receptor	KIT	P10721	CHEMBL1936	Kinase
Vascular endothelial growth factor receptor 3	FLT4	P35916	CHEMBL1955	Kinase

Tyrosine-protein kinase receptor FLT3	FLT3	P36888	CHEMBL1974	Kinase
Platelet-derived growth factor receptor alpha	PDGFRA	P16234	CHEMBL2007	Kinase
Kinesin-1 heavy chain/ Tyrosine-protein kinase receptor RET	RET	P07949	CHEMBL2041	Kinase
Tyrosine-protein kinase YES	YES1	P07947	CHEMBL2073	Kinase
Tyrosine-protein kinase BLK	BLK	P51451	CHEMBL2250	Kinase
Tyrosine-protein kinase JAK1	JAK1	P23458	CHEMBL2835	Kinase
Tyrosine-protein kinase HCK	HCK	P08631	CHEMBL3234	Kinase
Mixed lineage kinase 7	MAP3K20	Q9NYL2	CHEMBL3886	Kinase
Tyrosine-protein kinase Lyn	LYN	P07948	CHEMBL3905	Kinase
Serine/threonine-protein kinase Aurora-C	AURKC	Q9UQB9	CHEMBL3935	Kinase
Fibroblast growth factor receptor 4	FGFR4	P22455	CHEMBL3973	Kinase
Serine/threonine-protein kinase 10	STK10	O94804	CHEMBL398	Kinase

			1	
Tyrosine-protein kinase ABL2	ABL2	P42684	CHEMBL4014	Kinase
Serine/threonine-protein kinase 2	SLK	Q9H2G2	CHEMBL4202	Kinase
Tyrosine-protein kinase FRK	FRK	P42685	CHEMBL4223	Kinase
Tyrosine-protein kinase FGR	FGR	P09769	CHEMBL4454	Kinase
Ephrin type-A receptor 6	EPHA6	Q9UF33	CHEMBL4526	Kinase
Tyrosine-protein kinase BRK	PTK6	Q13882	CHEMBL4601	Kinase
Serine/threonine-protein kinase RIPK2	RIPK2	O43353	CHEMBL5014	Kinase
Serine/threonine-protein kinase B-raf	BRAF	P15056	CHEMBL5145	Kinase
Serine/threonine-protein kinase TNNI3K	TNNI3K	Q59H18	CHEMBL5260	Kinase
Epithelial discoidin domain-containing receptor 1	DDR1	Q08345	CHEMBL5319	Kinase
Eukaryotic translation initiation factor 2-alpha kinase 4	EIF2AK4	Q9P2K8	CHEMBL5358	Kinase

Citron Rho-interacting kinase	CIT	O14 578	CHEM BL557 9	Kinase
Serine/threonine-protein kinase 35	STK3 5	Q8T DR2	CHEM BL565 1	Kinase
Mitogen-activated protein kinase kinase kinase 7	MAP 3K7	O43 318	CHEM BL577 6	Kinase
Mitogen-activated protein kinase kinase kinase 2	MAP 3K2	Q9Y 2U5	CHEM BL591 4	Kinase
Mitogen-activated protein kinase kinase kinase 3	MAP 3K3	Q99 759	CHEM BL597 0	Kinase
Mitogen-activated protein kinase kinase kinase kinase 4	MAP 4K4	O95 819	CHEM BL616 6	Kinase
SPS1/STE20-related protein kinase YSK4	MAP 3K19	Q56 UN5	CHEM BL619 1	Kinase
LDL receptor	LDL R	P01 130	CHEM BL331 1	Membrane receptor
MAP kinase-activated protein kinase 2	MAP KAP K2	P49 137	CHEM BL220 8	Kinase
Poly [ADP-ribose] polymerase-1	PAR P1	P09 874	CHEM BL310 5	Enzyme
3-phosphoinositide dependent protein kinase-1	PDP K1	O15 530	CHEM BL253 4	Kinase
MAP kinase p38 alpha	MAP K14	Q16 539	CHEM BL260	Kinase

Nucleotide-binding oligomerization domain-containing protein 1	NOD 1	Q9Y 239	CHEM BL129 3222	Unclassified protein
Prostanoid EP1 receptor	PTG ER1	P34 995	CHEM BL181 1	Family A G protein-coupled receptor
Cholecystokinin A receptor	CCK AR	P32 238	CHEM BL190 1	Family A G protein-coupled receptor
6-phosphofructo-2-kinase/fructose-2,6-bisphosphatase 3	PFK FB3	Q16 875	CHEM BL233 1053	Enzyme
Serine/threonine-protein kinase mTOR	MTO R	P42 345	CHEM BL284 2	Kinase
PI3-kinase p110-alpha subunit	PIK3 CA	P42 336	CHEM BL400 5	Enzyme
Thrombin	F2	P00 734	CHEM BL204	Protease
HERG	KCN H2	Q12 809	CHEM BL240	Voltage-gated ion channel
Tyrosine-protein kinase SRC	SRC	P12 931	CHEM BL267	Kinase
Cathepsin K	CTS K	P43 235	CHEM BL268	Protease
Cathepsin S	CTS S	P25 774	CHEM BL295 4	Protease
Metabotropic glutamate receptor 5 (by homology)	GRM 5	P41 594	CHEM BL322 7	Family C G protein-coupled receptor
